# Intraoperative drug delivery to hindbrain tumours via an injectable hydrogel is well tolerated and confers survival benefit against atypical teratoid/rhabdoid xenografts

**DOI:** 10.1007/s13346-025-02034-0

**Published:** 2026-01-04

**Authors:** Cara Moloney, Phoebe McCrorie, Amr ElSherbeny, Harry Porter, Chiara Bastiancich, Hasan Slika, Aanya Shahani, Emre Derin, Esteban Velarde, Jackson Miller, John Theodore, Khushi Varshney, F. N. U. Ruchika, Hulya Bayraktutan, Umut Can Oz, Pam Collier, Simon M. L. Paine, Paul Handley, Keith Dredge, Grzegorz Wicher, Richard G. Grundy, Henry Brem, Karin Forsberg-Nilsson, Stuart J. Smith, Betty Tyler, Cameron Alexander, Ruman Rahman

**Affiliations:** 1 Children’s Brain Tumour Research Centre, School of Medicine, Biodiscovery Institute, University of Nottingham, Nottingham, UK; 2 Division of Molecular Therapeutics and Formulation, School of Pharmacy, University of Nottingham, Nottingham, UK; 3 Ex Vivo Cancer Pharmacology Centre of Excellence, School of Medicine, University of Nottingham, Nottingham, UK; 4 Aix-Marseille Université, Marseille, France; 5 Department of Neurosurgery, Johns Hopkins University, Baltimore, MD USA; 6 Department of Radiation Oncology, Johns Hopkins University, Baltimore, MD USA; 7 Department of Pharmaceutical Technology, Faculty of Pharmacy, Ankara University, Yenimahalle, Ankara, 06560 Türkiye; 8 Department of Neuropathology, Nottingham University Hospitals Trust, Nottingham, UK; 9 Zucero Therapeutics Ltd., Suite 1.11, Westlink Court, Darra, QLD Australia; 10 Department of Immunology, Genetics and Pathology and Science for Life Laboratory, Uppsala University, Uppsala, Sweden; 11 Science for Life Laboratory, Uppsala University, Uppsala, Sweden; 12 School of Medicine, Biodiscovery Institute, University of Nottingham, Nottingham, UK

**Keywords:** Local drug delivery system, Polymer hydrogel, Medulloblastoma, Atypical teratoid rhabdoid tumours

## Abstract

Intraoperatively applied local drug delivery systems (LDDS) offer a means of overcoming blood–brain barrier (BBB) impermeability. However, there is a paucity of LDDS development for paediatric tumours arising in the posterior fossa. Here we demonstrate applicability of an LDDS against medulloblastoma group 3 (G3 MB) and atypical teratoid/rhabdoid tumours (AT/RT), neoplasms associated with poor prognoses. A poly(ethyleneglycol)-poly(caprolactone)-poly(ethyleneglycol) (PECE) hydrogel loaded with chemotherapeutics identified as effective against primary G3 MB and AT/RT in vitro, was prepared as an injectable, biodegradable formulation. CHIR99021 (glycogen synthase kinase-3 inhibitor), ribavirin (guanosine analogue) and PG545 (heparanase inhibitor) were chosen based upon an inability to traverse the BBB. The hydrogel alone was well-tolerated, and drug-loaded hydrogel achieved > 1-month therapeutic release. Orthotopic xenograft studies against G3 MB and AT/RT indicated good tolerability to combined CHIR99021 and PG545 or combined CHIR99021 and ribavirin loaded loaded LDDS respectively. Median survival of AT/RT arms receiving XRT alone was comparable to CHIR99021- and ribavirin-loaded LDDS, with long-term survivors observed only in the latter arm, demonstrating a significant survival benefit. LDDS against cerebellar tumours using PECE offers a promising therapeutic alternative and the possibility of circumventing radiation-induced adverse effects for children impacted by these diseases.

**Graphical Abstract:**

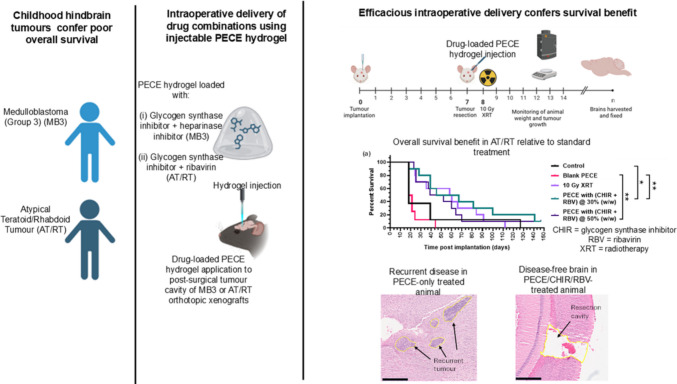

**Supplementary Information:**

The online version contains supplementary material available at 10.1007/s13346-025-02034-0.

## Intraoperative combination drug delivery using PEG-poly(caprolactone)-PEG hydrogel confers survival benefit to hindbrain tumours

### Background

Paediatric brain tumours arising in the posterior fossa within the hindbrain account for 54–70% of all childhood brain tumours [1], and include medulloblastoma (MB) and the majority of atypical teratoid/rhabdoid tumours (AT/RTs). MB is the most common malignant paediatric brain tumour and Group 3 MYC-amplified MB (G3 MB) confers the poorest prognosis with an overall 5-year survival below 50% [2]. Current standard treatment consists of maximal safe surgical resection with adjuvant chemotherapy and craniospinal radiation where feasible [3].

AT/RTs most commonly present in the first three years of life [4] and represent ~ 10% of primary brain tumours in patients under one year of age [5]. Median overall survival is ~ 13 months from diagnosis and dependent on the treatment administered [6]. AT/RTs are further classified by three broad distinctive molecular subgroups: sonic hedgehog (SHH); tyrosinase (TYR); and MYC proto-oncogene (MYC) [7].

Due to the lack of treatment consensus for G3 MB and AT/RT, the application of surgery as front-line therapy, and the need to avoid adverse neurocognitive and chronic sequelae conferred by radiotherapy (XRT), localised intraoperative drug delivery offers potential as a treatment option for these hindbrain tumour types. Additionally, the restrictive nature of the blood–brain barrier (BBB) limits the efficacy of some treatments due to their inability to access the brain following systemic injection [8–10]. As a result of this, local drug delivery systems (LDDS) have gained much attention to overcome poor BBB drug penetration, in principle leading to higher concentrations of drugs delivered to the target site and reduced off-target effects as a corollary [11, 12].

We previously reported on the intraoperative application of a LDDS, comprising a mouldable and temperature-sensitive poly(lactic-co-glycolic acid)-poly(ethylene glycol) (PLGA/PEG) paste loaded with etoposide and temozolomide (ETOP/TMZ), to the resection cavity of an orthotopic rat glioma model, whereby a significant increase in overall survival benefit was observed benchmarked against standard treatment [13]. Furthermore, when Olaparib (radiosensitiser) was combined with TMZ in the PLGA/PEG paste prior to administration of 10 Gy XRT, 57% of animals were long-term survivors [14]. However, whilst the application of a polymeric paste (via micro-spatula) was feasible in a preclinical rat brain resection model, much smaller surgical resection brain volumes hamper applicability to our previously developed mouse AT/RT and G3 MB orthotopic xenografts [15, 16]. Another promising type of LDDS involves the application of injectable hydrogels (HGs) to the tumour cavity following surgical resection, ensuring amenability for assessment in preclinical mouse xenograft brain models [17]. The injectable nature of HGs offers conformation to the irregular shapes of resection cavities, allowing for better contact between brain parenchyma harbouring residual tumour cells and the LDDS. We have previously reported the application of prodrug nanomedicine-based HGs against orthotopic glioblastoma models which were well tolerated, sustainedly delivered either gemcitabine or doxorubicin, and enhanced survival in several rodent models [18, 19].

Through the application of LDDS, drugs efficacious against relevant cancer cells in vitro but which were not clinically translated due to their inability to cross the BBB, can be revisited and repurposed, offering a distinct advantage over systemic chemotherapy. For example, we have demonstrated that PG545, a heparan sulphate mimetic, acts as a heparanase inhibitor which leads to a reduction of proliferation, migration and invasion of MB cell lines in vitro [20]. However, PG545 cannot cross the BBB due to its high molar mass of 2363.8 Da [21]. Similarly, CHIR99021 (CHIR), a glycogen synthase kinase-3 (GSK-3) inhibitor, reduced proliferation and self-renewal capabilities of MB cell lines in vitro but showed poor BBB permeability [22]. Finally, Ribavirin (RBV), a guanosine analogue which has been reported by us and others as an anti-viral, has shown promise against several brain cancer cell lines, including gliomas and AT/RT [15, 23, 24]. Intraperitoneal injection of RBV against an orthotopically implanted AT/RT patient-derived cell line conferred a significant increase in survival compared to a vehicle control. However, no long-term survivors were noted in this study and RBV was required to be administered daily.

In ovarian cancer, PG545 has recently been reported to induce DNA single- and double-strand breaks and impair homologous recombination repair by reducing RAD51 protein levels in an autophagy-dependent manner, thereby sensitising cells to poly (ADP-ribose) polymerase inhibitors [25]. GSK-3 inhibition results in increased expression of genes involved in serine/one-carbon metabolism, promoting metabolic vulnerabilities in lung and pancreatic cancer in vitro and in vivo [26, 27]. Protein synthesis inhibition by RBV has also been reported in acute myeloid leukaemia, oropharyngeal squamous cell carcinoma, and metastatic breast cancer [15]. RBV target engagement in several cancers is eIF4E, which is modulated through direct binding and competitive inhibition. Recent studies also report modulation of ERK, EZH2, and IMPDH upon RBV administration in high-grade gliomas [24, 28].

In this work, we describe the preparation and characterisation of an injectable biodegradable HG, composed of PEG-poly(caprolactone)-PEG (PEG-PCL-PEG) (or PECE), for application in the treatment of MB and AT/RT. PG545, CHIR and RBV were chosen as experimental and repurposed drug candidates for incorporation into PECE, predicated on their inability to cross the BBB. PECE co-polymers are thermosensitive [29], whereby the biomaterial behaves as a liquid at lower temperatures (4–20 °C), permitting syringe injection, and subsequently undergoes a non-linear viscosity increase (or gelation) upon reaching body temperature (37 °C), generating a matrix which conforms to the tumour resection cavity. Furthermore, PECE co-polymers are more amenable for intraoperative administration (via injection and subsequent gelation) to the small volumes of preclinical mouse xenograft brains, relative to thermosensitive paste formulations which require to be administered using a micro-spatula. Due to the challenges in achieving safe surgical resection in the mouse cerebellum, our study has assessed tolerability and efficacy against tumours xenografted into the mouse cortex. As a proof-of-concept, we demonstrate safe and efficacious application of a hydrogel-based LDDS against G3 MB and AT/RT neoplasms, which to the best of our knowledge, is the first report of a delivery system implanted directly in the tumour bed following surgical resection of both tumour types.

## Methods

### Preparation and characterisation of mPEG-b-PCL-HMDI-PCL-b-mPEG (PECE) hydrogel

PECE pentablock polymer was synthesized through a ring-opening polymerization reaction and characterized as previously reported [29]. Blank PECE hydrogels (HGs) were prepared through the dissolution of 200 mg of polymer in 1 mL PBS (20% w/w), using repeated heat cycles (50 °C), vortexing, and cool cycles (4 °C), until complete dissolution was achieved. For release in vitro studies, drug loadings of 1.5% (w/w) in a 20% (w/w) PECE HG were chosen to achieve release at a sufficient level to be quantifiable and to impact cell viability/metabolic activity following release of the drugs into cell culture media. To prepare dual drug loaded HGs at these concentrations, 1.2 mg each of CHIR99021 (CHIR) (Generon) and either PG545 (Zucero Therapeutics) or Ribavirin (RBV) (Stratech), were dissolved in 1 mL PBS with vortexing and sonication. 30 mg of PECE was added, and the solution was exposed to repeated heat/cool cycles until complete dissolution. The resultant solution was mixed with 1.8 mg each of CHIR and either PG545 or RBV and 170 mg of PECE and exposed to the heat/cool cycle until a homogeneous HG solution was obtained. Single drug loaded HGs were prepared using the same method, with the exclusion of CHIR, PG545 or RBV at both steps. The final drug loading concentrations of each agent in both single and dual loaded systems was *c.* 1.5% (w/w) in a 20% (w/w) PECE HG. A two-step process for loading the drugs into the HG was employed to ensure full solubility of the drugs in the LDDS. For in vivo studies, dual drug loadings of 30 or 50% (w/w) in a 20% (w/w) PECE HG were chosen to achieve an appropriate dose following local administration of the HG into the surgical resection cavity. HGs were prepared using the same method as above, with increased drug amounts added in each step. The higher drug loading did not quantitatively alter the gel formation of the LDDS.

The rheological behaviour of PECE HGs, with and without the incorporation of drugs at a loading of 1.5% (w/w) in a 20% (w/w) PECE HG was investigated using an Anton Paar MCR 302 Modular Compact Rheometer (Austria The influence of temperature on the storage modulus (G’), loss modulus (G"), and complex viscosity (i.e. the measure of the total resistance to flow as a function of angular frequency) was assessed by heating samples from 10 to 60 °C at a rate of 1 °C/min. Measurements were performed under controlled strain (1%) and frequency (1.0 Hz). The gelation temperature (T_gel_) was determined as the point at which G’ surpassed G". All rheological data were analysed using RheoCompass software (Anton Paar, Austria).

Scanning electron microscopy (SEM) was performed using a JEOL 7100 F scanning electron microscope (JEOL, UK) operating at an accelerating voltage of 15 kV. Blank PECE HGs were snap-frozen in liquid nitrogen and lyophilised. The resulting dried samples were mounted on aluminium stubs using double-sided carbon tape and sputter-coated with an 8 nm layer of iridium (Model: 150 T ES, Quorum, UK) and imaged.

### In vitro drug release

The release profiles of CHIR and either PG545 or RBV at drug loadings of 1.5% (w/w) from a 20% (w/w) PECE HG were recorded at 37 °C in PBS (pH = 7.4). 100 µL of drug loaded PECE (single or dual agent) was pipetted into a 2 mL centrifuge tube and set at 37 °C for 30 min before incubating in 1 mL PBS. At desired time intervals, the PBS was removed and replaced with a fresh aliquot to ensure sink conditions were maintained. Release of CHIR was quantified by High Performance Liquid Chromatography (HPLC) using a mobile phase gradient of H_2_O with 1% TFA and ACN (ACN content was maintained at 5% for 5 min followed by a linear increase to 95% over 10 min, a linear increase to 100% over 6 min, maintained at 100% for 4 min, before returning to the original conditions over 1 min), at a flow rate of 0.5 mL/min. A Zorbax C18 column (2.1 × 150 mm, 3.5 µm) was used with a VWD set to 280 nm for detection. 50 µL of sample was injected and a run time of 30 min was used. A representative chromatogram has been showed in Figure S3a, highlighting a retention time of *c.* 10.3 min.

Release of RBV was quantified by HPLC using a mobile phase consisting of 0.1% (w/v) Na_2_SO_4_ and 0.01% (v/v) H_3_PO_4_ in HPLC grade water at a flow rate of 0.5 mL/min. A Zorbax C18 column (2.1 × 150 mm, 3.5 µm) was used with a VWD set to 207 nm for detection. 50 µL of sample was injected and a run time of 6 min was used. A representative chromatogram has been showed in Figure S3b, highlighting a retention time of *c.* 1.8 min.

For PG545, 100 µL of the PG545 standards or release media were placed in quadruplicate into a 96-well plate and 20 µL of a 200 µg/mL stock of toluidine blue was added. A blank composed of PBS in place of the PG545 standard was also analysed. The plate was analysed on a TECAN Spark10M plate reader and the absorbance at 630 nm was recorded.

### In vitro cell culture and drug IC50 assessment

MB cell lines, D283, D341 and D425, were maintained in Minimum Essential Medium Eagle supplemented with 10% foetal bovine serum, with the addition of 1% non-essential amino acids for D425, at 37 °C in 5% CO2-humidified incubators. AT/RT cell lines, BT12, BT16, and BT37, were maintained in Dulbecco’s Modified Eagle Medium for BT12 and BT16, or RPMI medium for BT37, supplemented with 10% foetal bovine serum at 37 °C in 5% CO2-humidified incubators. The PrestoBlue™ Cell Viability assay was performed to assess metabolic activity of cells following exposure to PG545, CHIR and a dual treatment for the MB cell lines (concentration range of 0.3–22 µM for individual drugs; combination applied at a 1:1 molar ratio), or following exposure to RBV, CHIR and a dual treatment for AT/RT cell lines (concentration range of 1–1000 µM for individual drugs; combination applied at a 1:1 molar ratio). PG545 and CHIR have been previously reported as being effective as single agents against G3 MB cell lines [20, 22], while RBV has been reported as being effective against AT/RT cell lines [23]. Combinations of these drugs were investigated to assess the impact of a dual load LDDS.

Cells were seeded into 96 well plates at a density of 1 × 10^4^ (MB lines) or 5 × 10^3^ (AT/RT lines) cells/well and cultured for 24 h prior to 72-h exposure to treatments. Following this, the media was removed and replaced with 10% (v/v) PrestoBlue™ reagent diluted in PBS, the plate incubated for 30 min at 37 °C and the fluorescence was measured at 544/590 nm (ex/em) on a TECAN Infinite M Plex plate reader. Relative metabolic activity was calculated by setting normalised values from the negative control as 100%. IC50 values were calculated using GraphPad prism and dose response curves. For MB cell lines, 96 well plates were coated with poly-L-lysine (PLL) prior to beginning assays due to the semi-adherent nature of the cells. 40 µL of a 15 µg/mL PLL solution in H_2_O was added to each well and the plate was incubated at 37 °C overnight before washing with 50 µL H_2_O.

The D425 and BT12 cell lines were fLuc tagged for in vivo studies using a spinoculation method. Briefly, 1 × 10^6^ cells in the presence of 8 µg/mL polybrene and pLVX-Puro Vector containing Firefly luciferase and a Puromycin resistance gene (Clontech, Takara Bio Company, Otsu, Japan) were centrifuged for 2 h at 2000 × g, room temperature. The cell pellet was then resuspended in 1 mL fresh medium and span at 300 × g for 5 min, repeated twice. The viral load was assessed using Go-Stix kit, and transduced cells were selected by the addition of 10 µg/mL puromycin.

### In vitro drug activity following release from LDDS

Retention of CHIR, PG545 and RBV drug activity following release from PECE HGs was investigated against MB and AT/RT cell lines later utilised for in vivo experiments (D425 for CHIR and PG545, and BT12 for CHIR and RBV, respectively). Cells were seeded at a density of 5 × 10^4^ (D425) or 2.5 × 10^4^ (BT12) cells per well in a 24-well plate in 600 µL of respective media and incubated at 37 °C and 5% CO_2_ overnight. One hundred µL of each 20% (w/w) HG prepared at 1.5% w/w drug loadings were pipetted into Transwell ThinCert™ inserts (0.4 µm, transparent, Grenier-bio) in triplicate and set at 37 °C for 30 min before an additional 100 µL of media was added and placed into the 24-well plate and incubated for 72 h. For the BT12 cell line, a PrestoBlue™ assay was employed to assess metabolic activity as described above. For the D425 cell line, the suspension of cells was collected and stained with Trypan Blue, and the number of live and dead cells were manually counted to determine the viability of the cells.

### In vivo biocompatibility

Short-term tolerability studies were conducted at Aix-Marseille Université and were approved by the Animal Care and Use Committee (CE71, Aix-Marseille Université) and performed following the French national regulation guidelines in accordance with EU Directive 2010/63/EU. The studies were performed on the blank PECE HG using a previously reported method [30] on seven-week-old female athymic Nude-Foxn1nu mice (Envigo, Gannat, France). Mice were anesthetised with ketamine/xylazine (100 and 10 mg/kg intraperitoneal injection, respectively) and fixed on a stereotactic device, prior to the head being disinfected with an antiseptic solution (Vétédine® solution, Vetoquinol, Lure, France). Twenty-thirty µl of lidocaine (10 mg/ml, Aguettant, France) was injected subcutaneously onto the head and the eyes protected with an ophthalmic gel (Ocry-gel, TVM lab, Lempdes, France). An incision (3–5 mm) was made along the midline, and a burr hole drilled into the cranium above the right frontal region, 0.5 mm posterior and 2.1 mm lateral to the bregma using a high-speed drill (Tack Life Tools, USA; 0.8 mm diameter round end engraving burrs: Dremel, The Netherlands). Ten or 15 µl of HG were injected using a 30G insulin syringe at 2.5 mm or 3 mm depth from the outer border of the cranium, respectively. The skin was closed using a tissue adhesive (3 M Vetbond®, Sergy-Pontoise, France), and animals recovered under an infrared lamp. Animals were sacrificed humanely on day 15 post-injection, and brains fixed in 4% paraformaldehyde (Merck, Darmstadt, Germany) and sectioned.

### In vivo safety and efficacy

All animals were treated in accordance with the policies and guidelines of the Johns Hopkins University Animal Care and Use Committee. ARRIVE reporting guidelines were used for all animal work [31]. Animals were maintained in individually ventilated cages (Harlan Bioproducts) within a barriered unit, illuminated by fluorescent lights set to give a 12-h light–dark cycle (on 07.00, off 19.00), as recommended in the U.S. Public Health Service Policy on Humane Care and Use of Laboratory Animals. In vivo safety and efficacy studies of drug loaded PECE HGs against cortical orthotopic xenografts of D425 and BT12 cell lines, were conducted using NU/NU athymic mice (6–7 weeks old) (Charles River Laboratories). After confirming proper anesthesia, a burr hole was made 2–5 mm posterior and 1–2 mm lateral to bregma using a Stryker TPS universal drill until the dura was visible and subsequently opened. Next, 3 µL of firefly luciferase-tagged D425 (4.5 × 10^5^) cells or firefly luciferase-tagged BT12 (1.5 × 10^5^) cells suspended in their respective media were injected slowly over the span of one minute using a 26G Hamilton syringe.

Tumour burden was confirmed via bioluminescence-based imaging using an in vivo imaging system (IVIS Lumina III, Revvity, Waltham, MA) 7 and 8 days after implantation for the BT12 and D425 cohorts, respectively. For the imaging procedure, mice received a 0.2 mL intraperitoneal injection of a D-luciferin solution (15 mg/mL) (GoldBio, St. Louis, MO) and were induced in an isoflurane-based anaesthesia chamber. They were subsequently placed into the IVIS platform with their noses positioned inside the respective nose cones to guarantee continuous anaesthesia, and the images were acquired 5 min following the injection of D-Luciferin. Luminescence intensity was measured in the region of interest (the head of each), and the "total Flux" measurement was used to determine tumour burden. The mice were then stratified into the different treatment arms based on tumour burden, to ensure that the average and standard deviation for bioluminescence were equal among the groups." **Randomization**: Animals were assigned randomly (coin-flip) to treatment or control arms (1:1 ratio) for the efficacy study, and data collected per arm. **Blinding**: The allocation of animal groups was blinded to care staff where possible, to ensure animal husbandry was invariable for all experimental animals. **Sample size**: G* Power free online software was used to calculate effective sample size. A sample size of *n* = 10 per treatment arm (where possible due to sufficient tumour uptake) was determined by the Wilcoxon-Mann–Whitney test as appropriate for in vivo efficacy assessment. This was based on 80% power (5% significance; two-sided difference of means), where a standardized effect size (signal/noise ratio of 1.6) was estimated from our previous tolerability studies comparing each individual treatment arm for brain tumour localized delivery of temozolomide and etoposide, versus surgery only control [13]. **Rules for stopping data collection**: Animal weight loss/food avoidance and/or neurological deficit were deemed end-points due to treatment-related adverse effects, at which stage, animals were humanely euthanized. **Data inclusion/exclusion criteria**: Inclusion criterion for the in vivo study was an animal weight range of 20–25 g. Exclusion criterion was adverse complications due to surgery.

Mice in the D425 cohort were allocated to the following groups: [1] resection + radiation therapy (*n* = 3), [2] resection + 10 µL PECE HG with CHIR-99021 and PG545 (1:1 ratio) loaded at a total drug content of 50% (w/w) (*n* = 6), [3] resection + radiation therapy + 10 µL PECE HG with CHIR-99021 and PG545 loaded at 50% (w/w) (*n* = 5). Mice in the BT12 cohort were allocated to the following treatment groups: [1] resection only (*n* = 8), [2] resection + 10 µL blank PECE HG (*n* = 8), [3] resection + radiation therapy (*n* = 10), [4] resection + 10 µL PECE HG with CHIR-99021 and RBV loaded at 30% (w/w) (*n* = 10), [5] resection + 10 µL PECE HG with CHIR-99021 and RBV loaded at 50% (w/w) (*n* = 10). Resection was performed using a glass pipette connected to continuous suction under the guidance of a Zeiss surgical microscope (Zeiss OPMI 6-CFC surgical microscope). Radiation therapy (single 10 Gy CT-guided X-ray radiation dose) was delivered the day after the mice recovered from surgery using an XSTRAHL Small Animal Radiation Research Platform (SARRP). The mice were subsequently weighed twice weekly and imaged to assess tumor burden.

### Histology and immunohistochemistry

For Haematoxylin and Eosin staining, paraffin embedded slides were deparaffinised before being treated sequentially in 100, 90, 70, 50% IMS, water, Harris haematoxylin and eosin (Surgipath, UK). Stained sections were dehydrated, mounted with DPX mounting medium (Sigma) and imaged on a NanoZoomer®-SQ (Hamamatsu). For Ki67 (Abcam, ab16667) and CD68 (Abcam, ab283654) immunohistochemical staining, paraffin embedded slides were treated following the recommendations of the manufacturer. The primary antibody diluted in 30% (v/v) NGS in PBS (1/50 for Ki67, 1/100 for CD68) was added to the slides and incubated at 4 °C overnight. The slides were then incubated with the secondary antibody for 30 min, followed by DAB solution for a further 5 min. The samples were counterstained with haematoxylin, dehydrated and mounted with DPX mounting medium (Sigma). Apoptosis was determined using a terminal deoxynucleotidyl transferase mediated deoxyuridine triphosphate nick end labelling assay, or TUNEL assay, following the manufacturer’s instructions (Oncor, Boehringer Mannheim, Germany). Slides were imaged on a NanoZoomer®-SQ and positive staining was quantified in the area defined as the tumour/cavity and in the infiltrative margin (designated as within 500 µm of the tumour/cavity) using QuPath-0.4.3.

### Statistical analysis

In vitro cytotoxicity results are reported as the inhibitory concentration 50% (IC50) for each cell line given as the mean and standard error of the mean (SEM) for three independent experiments, plotted relative to the percent viability of vehicle-normalized untreated cells. Statistical analysis was carried out using an ordinary two-way ANOVA with Tukey’s multiple comparisons test (or one-way ANOVA for incubation of drug loaded HGs with cells), where differences were considered significant when **** p < 0.0001, *** p < 0.001, ** p < 0.01, * p < 0.05. Instances where no statistical significance was noted are not indicated in the Figures.

Overall survival (OS) analyses were performed using GraphPad Prism (v10.2). OS was calculated from time of tumour implantation to death from any cause. Kaplan–Meier survival curves with significance levels determined by the log-rank test were constructed by univariate analyses. P values < 0.05 were deemed statistically significant.

## Results

### Synthesis and characterisation of in situ gelling injectable polymer drug delivery matrix

**Fig. 1:**
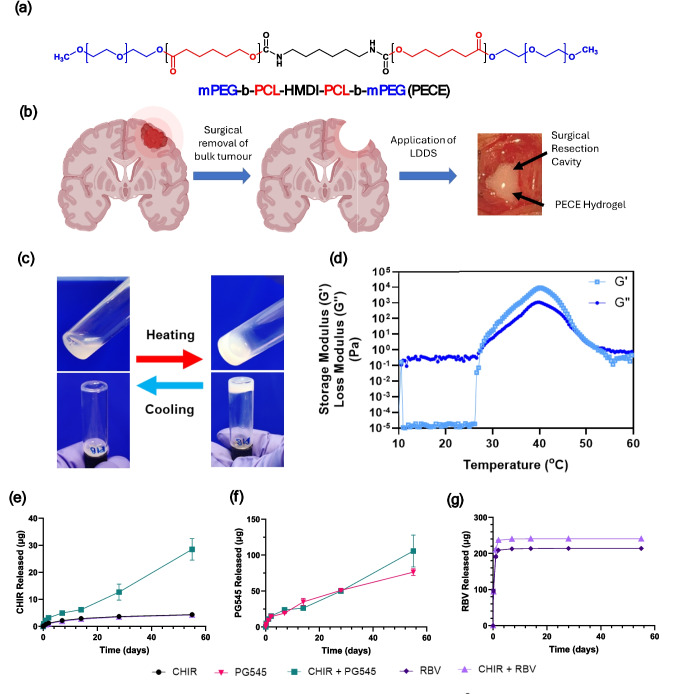
Synthesis and characterisation of PECE HG. (**a**) Chemical structure of mPEG-b-PCL-HMDI-PCL-mPEG (PECE) polymer employed in this work. (**b**) Schematic representation of the application of a LDDS following surgical tumour resection. (**c**) Visual representation of the reversible gelation of PECE upon heating or cooling. (**d**) Rheological evaluation of PECE HG prepared at 20% (w/w) showing effect of temperature on the storage and loss modulus. Recorded release plots of (**e**) CHIR, (**f**) PG545, and (**g**) RBV from single or dual drug loaded PECE HGs. Release was recorded from 50 µL HG aliquots loaded at concentrations of 1.5% (w/w) of each drug and incubated in PBS (pH = 7.4) at 37 °C (*n* = 3, release reported as average ± S.D)

A biodegradable, amphiphilic PECE pentablock copolymer (mPEG-b-PCL-HMDI-PCL-b-mPEG) (Fig. 1a) was synthesised and characterised through a two-step process as previously reported by us [32] in preparation for post-surgical LDDS application (Fig. 1b). We have previously demonstrated that this polymer forms injectable HGs which can be passed through a 29G needle easily, with no impact on gelling properties [32]. To examine the phase transition temperature of both blank PECE and drug-loaded PECE HGs at 20% w/w concentration, the test-tube-inversion method was employed, as depicted in Fig. 1c. At 20 °C, PECE solutions were free-flowing, but upon increasing the temperature to 37 °C, a sol–gel transition occurred. Dynamic rheological experiments (Fig. 1d) showed that at temperatures below 20 °C, PECE HGs (20% w/w) displayed low storage and loss moduli (G’ and G") values (< 1 Pa), indicative of good flow and injectability properties. As the temperature increased, a gelation point (T_gel_) at 27 °C was identified (G’ > G"), and by 37 °C, the G’ value increased by > 4 orders of magnitude, demonstrating the formation of a physically crosslinked HG. The incorporation of the drugs of interest in this work, CHIR and PG545, or CHIR and RBV, at concentrations of 1.5% (w/w) of each drug, led to an approximate 0.5–1 °C increase in T_gel_, likely caused by the disturbance of the original compact arrangement of PECE micelles (Figure S1a and b). Scanning electron microscopy (SEM) images revealed that PECE hydrogels exhibited a porous network structure after dehydration from solution (Figure S2).

### PECE HG enables loading, co-loading and release of drug candidates

Release profiles of CHIR, PG545 and RBV from single or dual loaded PECE HGs were next determined, both as overall amount of drug released and as a percentage of the total drug loaded into HGs (Fig. 1e–g). Loadings of 1.5% (w/w) of each drug were employed for these experiments as this resulted in sufficient amounts of drug being released for quantitation without depleting drug stocks. Release of CHIR release was sustained up to at least 8 weeks (4.3 ± 0.02 µg cumulative release) (Fig. 1e and Figure S4a and b), and the addition of RBV into CHIR loaded PECE HGs did not have a marked impact on CHIR release profiles, with comparable release observed (2.7 ± 0.03 and 4.2 ± 0.02 µg of CHIR released after 2 and 8 weeks, respectively). However, incorporation of PG545 into CHIR loaded PECE HGs resulted in elevated CHIR release, with *c.* 6.2 ± 0.13 and 28.5 ± 4.0 µg released after 2 and 8 weeks, respectively. For PG545 loaded HGs, a steady, sustained release of drug was noted up to 8 weeks of a higher magnitude than that from the CHIR loaded (Fig. 1f and Figure S4c). The inclusion of CHIR into PG545 loaded HGs resulted in comparable release following 2 weeks of incubation (23.8 ± 1.9 and 19.1 ± 0.6 µg of PG545 released, with and without the inclusion of CHIR, respectively). However, there was higher release from PG545 HGs co-loaded with CHIR (105.8 ± 22.2 µg) after 8 weeks of incubation when compared to HGs loaded with PG545 alone (76.4 ± 4.7 µg). Conversely, the release of RBV from the gels (Fig. 1g and Figure S4d and e) followed a burst profile, with 190.0 ± 5.3 µg released within the first 24 h of incubation when RBV was loaded as a single agent, with little further change after 8 weeks (214.0 ± 0.03 µg released). The inclusion of CHIR into RBV loaded HGs resulted in a similar release profile to that of RBV alone.

### Candidate drugs retain cytotoxic activity following release from PECE HGs in multiple cell lines

**Fig. 2:**
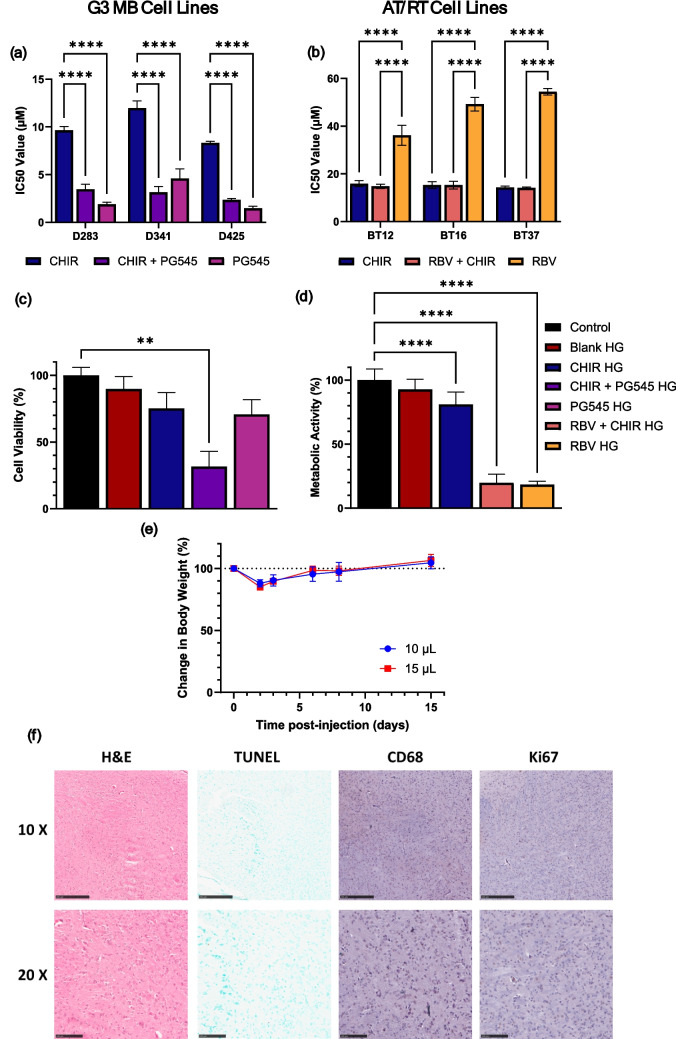
Effects of blank and drug containing PECE HGs in G3 MB and AT/RT cell line in vitro and blank HG in vivo. (**a**-**b**) Cytotoxicity testing of candidate drugs against a panel of G3 MB and AT/RT cell lines following 72 h of incubation. Recorded IC50 values of (**a**) CHIR, PG545 and 1:1 ratio of CHIR/PG545 combination against G3 MB cell lines D283, D341 and D425; and (**b**) CHIR, RBV and 1:1 ratio of CHIR/RBV combination against AT/RT cell lines BT12, BT16 and BT37 (*n* = 3, IC50 values reported as average ± SEM). Biocompatibility of blank PECE HG and retention of cytotoxicity of drugs following 72-h release from HG for (**c**) D425 and (**d**) BT12 (*n* = 3, cell viability and metabolic activity values reported as average ± S.D). (**e**) Change in mouse body weight following injection of 10 (*n* = 3) or 15 µL (*n* = 2) of 20% (w/w) PECE HG using a 30G insulin syringe at 2.5 mm or 3 mm depth from the outer border of the cranium, respectively. (**f**) Representative images of brain sections following treatment with 15 µL of 20% (w/w) PECE HG that have been stained (from left to right) with H&E, TUNEL assay, CD68 and Ki67. Top row: 10 X magnification, scale bar = 250 µm; Bottom row: 20 X magnification, scale bar = 100 µm

In vitro cytotoxicity of compounds as both single and dual agents was investigated in a panel of patient derived G3 MB and AT/RT (MYC and TYR subtypes) cell lines. Dual drug combinations for G3 MB were chosen in this study to firstly demonstrate the capability of the PECE HG to delivery multiple drugs representing distinct chemistries, towards development of a drug delivery platform technology that is well-suited to target heterogenous disease. Specifically, our choice for the combination of PG545 and CHIR for the treatment of G3 MB was pre-selected based on previous Group 3 MB studies by us and others [20, 22], whereby two distinct mechanisms of action (heparanase and GSK-3 inhibition respectively) could be assessed (Figure S5). Of the G3 MB cell lines investigated, D425 represents the cell line used to generate an orthotopic MB model, as this cell line conferred the most robust and reproducible uptake rate upon orthotopic injection. CHIR and PG545, alone and in combination, conferred IC50 values of 8.3 ± 0.3, 1.5 ± 0.4 and 2.3 ± 0.3 µM, respectively, against D425. There was a significant decrease in IC50 between PG545 and the combination across all cell lines (p < 0.0001), and while there was a slight increase in IC50 for the combination as compared to CHIR, this change is not significant and the impact of the combination on the cell lines was additive. Representative IC50 curves are shown in Figure S5a, with respective values reported in Fig. 2a and Figure S5c. The additional MYC-amplified G3 MB cell lines investigated (D283 and D341) demonstrated similar results with comparable IC50 values (Fig. 2a and Figure S5c).

For the AT/RT cell lines, BT12-MYC represents the cell line used to generate an orthotopic AT/RT model, as this cell line conferred the most robust and reproducible uptake rate upon orthotopic injection. Our choice for RBV in combination with CHIR was similarly based upon an aim to demonstrate delivery of distinct drug chemistries using PECE HG against heterogenous disease, and our previous identification of RBV as an AT/RT treatment candidate [15]. As we observed greater potency of CHIR against all three AT/RT cell lines tested relative to RBV, as shown in Figure S5, this drug combination was chosen. Similarly to drugs tested on the G3 MB panel, CHIR, RBV alone and in combination, conferred low IC50 values of 15.9 ± 2.2, 36.2 ± 7.3 and 14.8 ± 1.5 µM, respectively, against BT12 (Figure S5b, Fig. 2b and Figure S5d). The additional AT/RT cell lines investigated (BT16-MYC and BT37-TYR) demonstrated comparable IC50 values (Fig. 2b and Figure S5d).

Similarly to the G3 MB cell lines, there was a significant decrease in IC50 between RBV and the combination across all cell lines (p < 0.0001), and no significant change in IC50 for the combination as compared to CHIR, the impact of the combination on the cell lines was additive.

To ensure that drug-induced cytotoxicity was retained following incorporation and release from PECE HGs, cell lines applicable for in vivo models, (D425 and BT12 for G3 MB and AT/RT, respectively), were incubated with single and dual drug loaded HGs in Transwell® inserts for 72 h and metabolic activity determined. Blank HG did not significantly impact cell viability relative to the untreated control (89.6 ± 16.1%, ns), indicating cytocompatibility of the biomaterial (Fig. 2c). Incubation of the D425 cell line with HGs loaded with either CHIR or PG545 resulted in no significant decrease in cell viability (75.2 ± 20.3 and 70.7 ± 19.0% for CHIR and PG545, respectively). Importantly, the dual CHIR and PG545 loaded HG conferred a significant decrease in cell viability following 72 h drug exposure (31.5 ± 19.7%, p < 0.01), confirming that D425 cytotoxicity induced by CHIR and PG545 in combination, is unaffected by incorporation within, and release from, the PECE HG.

For the BT12 cell line, blank HG did not significantly alter metabolic activity (92.6 ± 8.0%), (Fig. 2d) whereas the drug loaded HG significantly impaired metabolic activity relative to untreated control (81.0 ± 9.6, 18.4 ± 2.7 and 19.8 ± 6.6% for CHIR, RBV and dual loaded CHIR and RBV HG respectively; p < 0.0001 for all comparisons to untreated control). These data confirmed that CHIR and RBV alone and in combination were released from PECE HGs and retained activity against BT12 cells.

### PECE HGs do not induce apoptosis, inflammation or proliferation in non-disease brains

To confirm in vivo biocompatibility of the PECE HG, mouse body weight was monitored over the course of 15 days post-injection of 20% (w/w) blank HG (Fig. 2e). Following an initial decrease in the 48 h following treatment, mouse body weight was recovered and retained up to 15 days post-injection, indicating HG biocompatibility following injection in the brain. H&E staining demonstrated no obvious change in brain structure adjacent to the injection site. Negative staining following a TUNEL assay indicated no instances of apoptosis. Additionally, the absence of positive staining for markers of inflammation (CD68) and proliferation (Ki67) demonstrates that the HG was well tolerated (Fig. 2f).

### G3 MB orthotopic xenograft tolerability to PECE drug-loaded HG

**Fig. 3:**
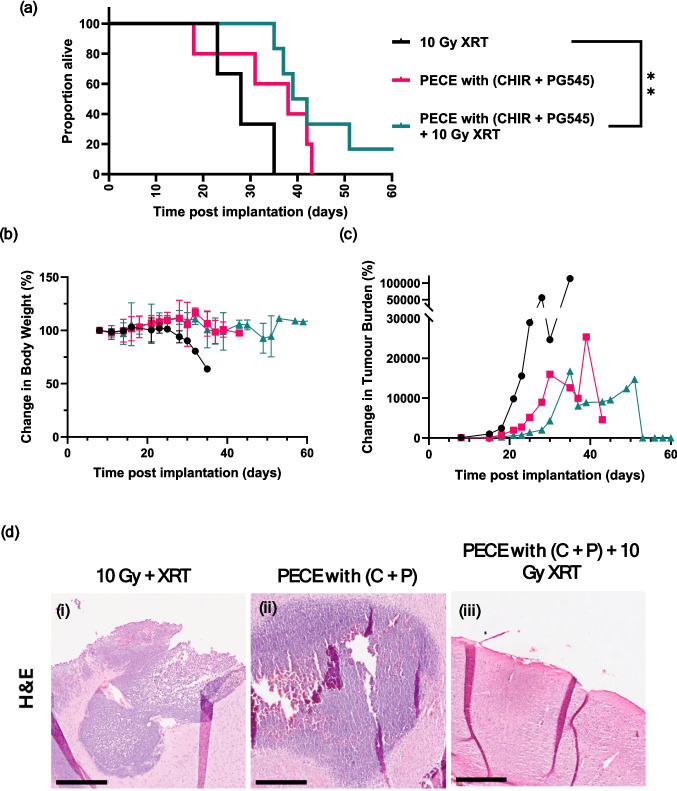
In vivo tolerability of locally delivered drug-loaded PECE HG in orthotopic G3 MB patient derived xenografts. (**a**) Kaplan–Meier overall survival plots of mice implanted with D425 G3 MB cells, which following surgical resection were treated with 10 Gy XRT (*n* = 3), 10 µL of PECE loaded with CHIR/PG545 at 50% (w/w) with (*n* = 6) or without (*n* = 5) the addition of 10 Gy XRT. (**b**) Recorded average change (%) in mouse body weight per treatment over the course of experiment, highlighting a decrease in body weight (after 25 days) in the group treated with XRT alone, and tolerability of CHIR and PG545 loaded PECE HGs (mean ± S.D). (**c**) Recorded average change (%) in tumour burden (as measured by IVIS imaging of fLuc tagged D425 cells) over the course of experiment (mean only, see Figure S6 for mean ± S.D). (**d**) Representative H&E staining of brain tissue of the longest survivor in each group (5X magnification, scale bar = 500 µm): (i) 10 Gy XRT, day 35; (ii) PECE loaded with (CHIR + PG545), day 43; and (iii) PECE loaded with (CHIR + PG545) and 10 Gy XRT, day 147 (LTS)

A pilot in vivo study was next undertaken using PG545 and CHIR loaded PECE HG against an orthotopic xenograft model of D425 G3 MB to assess tolerability. Here, a higher drug loading in the HG was employed compared to the drug release experiments to achieve a therapeutic concentration within the parenchyma following release from the LDDS. At these drug loading concentrations, the HG retained its desired thermoresponsive properties, was injectable at room temperature, formed a gel at body temperature, and retained the shape of the resection cavity (see Fig. 1b). Immunocompromised mice implanted with D425 cells underwent surgical resection of macroscopic tumour followed by treatment. 10 µL was chosen as the injection volume as this was sufficient to fill the cavity following tumour resection (Fig. 1b). Animals were monitored over the course of 60 days, with no adverse physical or neurological side-effects observed. The survival plot from this experiment is shown in Fig. 3a, with additional mean and median survival information reported in Table 1. At 60 days, one survivor remained in the experimental group treated with drug loaded HG and adjuvant XRT, which was then monitored further to assess the long-term safety. The median survival of groups treated with 10 Gy XRT, 10 µL of 20% (w/w) PECE loaded with combined CHIR/PG545 at 50% (w/w) alone or with adjuvant 10 Gy XRT, were 28.0-, 38.0- and 40.5-days post tumour implantation, respectively. The group treated with drug loaded HG and XRT exhibited a statistically significant increase in survival relative to the group treated with XRT alone (p < 0.01), with one animal surviving up to 147 days post tumour implantation. In the XRT treated group, a large decrease in mouse body weight was observed by day 35 (63.8%, Fig. 3b) which coincides with a large increase in tumour burden (1.1 × 10^5^%, Fig. 3c), suggesting the ineffectiveness of XRT alone in preventing tumour growth. For the groups treated with drug loaded HG, either with or without the addition of XRT, the recorded mouse body weight remained broadly constant, and the increase in tumour burden did not reach the same magnitude as that of the XRT only group (1.2 × 10^4^ and 1.7 × 10^4^%, respectively, on day 35), indicating that the drug loaded HG impacted tumour growth.

**Table 1:** Summary of median and mean overall survival in MB orthotopic xenografts (D425) treated with PECE HGs loaded at 50% (w/w) CHIR and PG545, with and without radiotherapy

Treatment following surgical resection	*n*	Mean survival (days)^a^	Median survival (days)	SEM	LTS	%LTS
10 Gy XRT	3	28.7	28.0	3.5	0	0.0
PECE with (CHIR + PG545)	5	34.4	38.0	4.6	0	0.0
PECE with (CHIR + PG545) + 10 Gy XRT	6	58.5	40.5	17.8	1	16.7

^a^Estimation is limited to the longest survival time if censored

Histological analysis was also undertaken to assess treatment impact at a cellular level in the resection cavity, recurrent tumour and surrounding parenchyma, of the longest survivor in each group (35-, 43- and 147-days post tumour implantation for groups treated with XRT, drug loaded HG and drug loaded HG with XRT, respectively). H&E staining demonstrated that when XRT or drug loaded HG was applied individually as treatments, a large tumour was present in the longest survivor of each group (Fig. 3d(i)and (ii)). In particular, the XRT treated animal exhibited an infiltrative tumour which had spread diffusely into surrounding brain parenchyma. Conversely, the combination of the two treatments resulted in the absence of tumour and no evidence of residual disease in the LTS (Fig. 3d (iii)).

### In vivo tolerability and efficacy of drug-loaded PECE HG for treatment of AT/RT

**Fig. 4:**
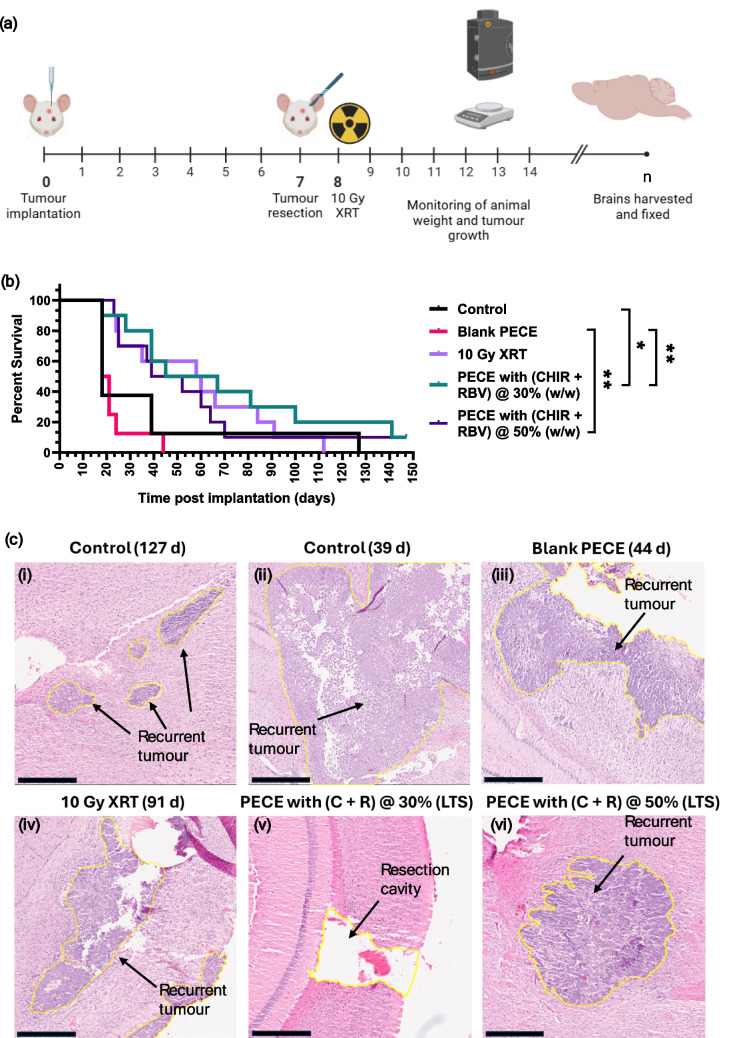
In vivo efficacy of locally delivered PECE loaded with CHIR and RBV in orthotopic AT/RT patient derived xenografts. (**a**) Schematic representation of the in vivo efficacy schedule. (**b**) Kaplan–Meier overall survival plots of mice implanted with BT12 cells, which following surgical resection were treated as follows: no treatment (control), 10 µL of blank PECE (20% w/w), 10 Gy XRT, 10 µL of PECE loaded with combined CHIR/RBV at 30 or 50% (w/w). Animals alive at termination of experiment after 147 days post tumour implantation were deemed LTS. **(c)** Representative H&E staining of longest survivor in each treatment group highlighting the presence of recurrent tumour or resection cavity (in yellow): (**i**) control day 127; (**ii**) control day 39; (**iii**) blank PECE day 44; (**iv**) 10 Gy XRT day 91; (**v**) PECE loaded with combined CHIR/RBV at 30% (w/w) day 147 (LTS); and (**vi**) PECE loaded with combined CHIR/RBV at 50% (w/w) day 147 (LTS). All images taken at 5X magnification, scale bar = 500 µm

After confirming the feasibility and tolerability of the PECE drug loaded HG to a surgical resection G3 MB model, we progressed towards a larger tolerability and therapeutic study using a resection model of an AT/RT patient derived orthotopic xenograft (BT12) as an exemplar testbed, due to the more robust tumour uptake rate in this model. Mice which had been implanted with BT12 xenografts underwent surgical resection of macroscopic tumour, followed by treatment (see Fig. 4a). Similar to the G3 MB model, animals were monitored for adverse physical and neurological side-effects over the course of the study, with no such effects observed, confirming tolerability. Across all treatment groups, mouse body weight remained broadly constant, with a slight increase observed in the control group at later timepoints (Figure S7a). Following 60 days post tumour implantation, the average increase in tumour burden for the XRT treated group was higher than that of both drug loaded HG groups (4.3 × 10^4^% for XRT group as compared to 1.5 × 10^4^ for HGs loaded with either 30 or 50% (w/w) of CHIR and RBV), indicating that drug loaded HGs impacted tumour growth (Figure S7b). Furthermore, a decrease in tumour burden towards the end of the experiments was noted for the remaining animals in the control and drug loaded HG groups and corresponds to the small tumours observed by histology.

The survival plot from AT/RT efficacy study is shown in Fig. 4b, with additional mean and median survival information reported in Table 2. The median survival of groups treated as control, or with blank PECE, 10 Gy XRT, 30% (w/w) PECE loaded with combined CHIR/RBV or 50% (w/w) PECE loaded with combined CHIR/RBV, were 18.0-, 19.5-, 59.0-, 56.0- and 45.5-days post tumour implantation, respectively. A significant increase in median survival was observed for animals treated with PECE HG loaded with combined CHIR/RBV at 30% (w/w) relative to the control group (p < 0.0232). The no treatment control group contained one outlier which survived for 127 days post-implantation, while the median survival was 18.0 days. This impacted the statistical analysis of the data; nevertheless, the blank HG control group can also be used as a comparator for drug treatment groups. There was a significant increase in median survival for both the 30 and 50% (w/w) combined CHIR/RBV loaded PECE HGs (56.0 and 45.5 days, respectively) as compared to the blank PECE HG group (p < 0.01 for both comparisons). Comparable survival was observed between groups treated with 10 Gy XRT alone, and groups treated with either 30 or 50% (w/w) combined CHIR/RBV loaded PECE HG, with no significance noted. Recorded mean survival also offers valuable insight into the efficacy of the treatments as it accounts for instances where long term survivors (LTS) are noted. The mean survival of groups treated as control, or with blank PECE, 10 Gy XRT, 30% (w/w) PECE loaded with combined CHIR/RBV or 50% (w/w) PECE loaded with combined CHIR/RBV, were 36.9-, 22.8-, 57.3-, 70.5- and 54.2-days post tumour implantation, respectively. These results mirror that of the median survival but emphasise the presence of the LTS in both drug loaded HG treated groups.

**Table 2:** Summary of median and mean overall survival in AT/RT (BT12) orthotopic xenografts treated with PECE HGs loaded at 30 or 50% (w/w) with combined CHIR and RBV

Treatment following tumour resection	n	Mean survival (days)^a^	Median survival (days)	SEM	LTS	%LTS
Control	8	36.9	18.0	13.3	0	0
Blank PECE	8	22.8	19.5	3.1	0	0
10 Gy XRT	10	57.3	59.0	10.1	0	0
PECE with (CHIR + RBV) @ 30% (w/w)	10	70.5	56.0	14.5	1	10
PECE with (CHIR + RBV) @ 50% (w/w)	10	54.2	45.5	11.6	1	10

^a^Estimation is limited to the longest survival time if censored

Histological analysis was undertaken on the longest survivor in each group, 127-, 44- and 91-days post tumour implantation for groups treated with surgery only, blank HG or XRT, respectively, with one LTS (survival up to 147 days post tumour implantation) evident for both the 30 and 50% drug loaded HGs. An additional animal from the control group was included in the analysis which was more representative of the median survival of the group (39-days post tumour implantation). H&E staining was undertaken, and images focusing on the tumour/cavity region are shown in Fig. 4c, along with images of whole brain sections shown in Figure S8a. The size of each tumour was quantified and reported in Figure S8b. The tumours in the control group display areas of 0.3 and 7.4 mm^2^, for the animals that survived 127 and 39 days, respectively. The tumour size in the blank HG (44 days), XRT treated (91 days), and 30% and 50% drug loaded HG (both LTS, 147 days) was 3.1, 1.0, 0.0 and 1.3 mm^2^, respectively. In the blank HG group, the tumour had spread into the ventricle, resulting in a blockage and large expansion as indicated in Figure S8a (iii). A haemorrhage was evident in the brain from the XRT treated group, in addition to many infiltrative cells (Figure S8a (iv)). The gross tumour morphology evident in 50% drug loaded HG is distinct from that of other treatment groups, whereby circumscribed tumour remains relatively localised, in contrast to other groups where the tumour is more diffuse (Figure S8a(vi)).

**Fig. 5:**
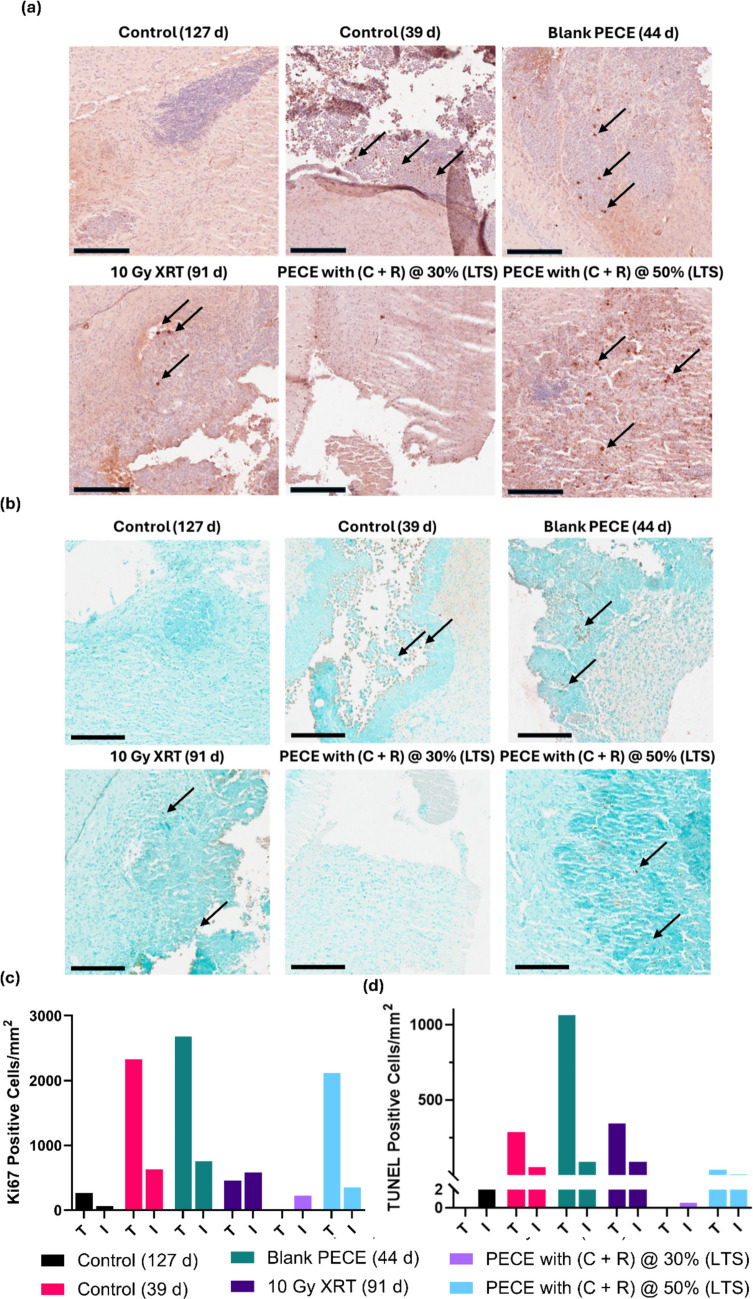
AT/RT post-sacrificial histological analyses of PECE drug-loaded HG. Representative (**a**) Ki67 (proliferation marker), and (**b**) TUNEL (apoptosis) IHC staining of longest survivor in each treatment group, with some incidences of positive staining indicated with black arrows. All images taken at 20X magnification, scale bar = 100 µm. (**c**) Quantification of the number of cells positively stained for (**c**) Ki67 and (**d**) TUNEL per mm^2^ of tissue in the tumour/cavity (T) and the surrounding healthy tissue up to 500 µm from the tumour cavity, denoted the infiltrative margin (I), for all treatment groups. PECE with (C + R) denotes HG loaded with combined CHIR/RBV

Ki67 staining was also undertaken (Fig. 5a) and the number of cells positively stained in both T and I were quantified (Fig. 5c). The control animal (which best represents the median survival of the group) and the longest survivor in the blank HG group, exhibited the highest levels of positive Ki67 staining, with 2326 and 2679 positive cells/mm^2^, respectively. A decrease in positive staining was observed with increased survival as XRT-treated, the outlier in the control group, and the drug loaded HG at 30%, exhibited Ki67 positive tumour staining of 458, 265 and 0 cells/mm^2^, respectively. The 50% drug loaded HG was an outlier to this trend with 2118 positive cells/mm^2^ of tumour, even though this was an LTS. With TUNEL staining (Fig. 5b and d), the blank HG exhibited the highest levels of positive staining (1065 cells/mm^2^) in the tumour region, followed by the control animal (39-day survival), and the XRT treated animal (286 and 346 cells/mm^2^, respectively). Low levels of positive staining were observed for the drug loaded HG at 50% (35 cells/mm^2^). There was an absence of positive staining in both the small tumour present in the longest survivor in the control group (127 days) and for the LTS from the 30% drug loaded HG group.

## Discussion

LDDS offer several potential advantages over systemic administration in the treatment of brain tumours by enabling BBB bypass and precise delivery of therapeutics to the resection cavity immediately after surgery [17]. In the current study, our focus was on the application of a LDDS against paediatric brain tumours arising in the posterior fossa, specifically G3 MB and AT/RT. A panel of patient derived cell lines were selected for both in vitro and in vivo evaluations of our LDDS, resulting in the need for an immunocompromised mouse model to permit xenograft tumour uptake. To increase biodistribution and flexibility of delivery, we developed an injectable drug loaded HG (PECE) for which the volume and dose can be controlled at smaller resection cavity volumes. PECE is an excellent candidate HG due to facile synthesis [29], biodegradability [33] and cytocompatibility [34]. We have previously reported on the degradation of this HG in PBS, with a *c.* twofold decrease in molar mass noted following 90-day incubation [32], with a faster degradation rate expected in the post-surgical section cavity due to the presence of esterases in cerebrospinal fluid [35]. Additionally, PECE is thermosensitive, behaving as a liquid at room temperature and gelling at 37 °C (Fig. 1c). This formulation allows for direct injection into a tumour cavity at room temperature, with subsequent gelation in contact with brain parenchyma and retention in the fullness of the cavity such that the whole resection surface can be addressed (Fig. 1b).

As brain stiffness is reported to be between 1–3.5 kPa when tested in the frequency range of 50–60 Hz [36], blank PECE HG was prepared at 20% (w/w), whereby a stiffness value of 2.3 kPa was recorded at 37 °C and at a frequency of 60 Hz (Fig. 1d), indicating that our LDDS has comparable stiffness to that of the brain. Importantly, soft HGs with a comparable stiffness to that of our blank PECE HG (2 kPa) did not significantly alter the tumourgenicity of the glioma lines in vivo, indicating the suitability of our LDDS [37].

The rationale for co-loading therapeutics into the HG was developed following initial scoping experiments which demonstrated varying release kinetics attributable to the chemical properties of the target drug molecules. Dual drug loading into the PECE HG was shown to be advantageous whereby incorporation of PG545 and CHIR in a PECE HG resulted in accelerated and overall higher release of CHIR. We attribute the difference in release of CHIR when co-loaded with other drugs, to the markedly different structures of the agents. PG545 has both a polysulfated sugar region and a rigid cholestanyl unit, enabling it to associate with CHIR via induced electrostatic interactions at the basic CHIR pyridyl and secondary alkyl amine groups, but also via hydrophobic interactions between the aromatic groups in CHIR and the cholestanyl tail, leading to solubilisation of CHIR in the HG in PG545-rich regions and in turn, enhanced partitioning of CHIR-PG545 adducts from the gel into the aqueous release media (Fig. 1e). Similarly, both PG545 and RBV release are marginally elevated when CHIR is incorporated into the HG (Fig. 1f-g respectively). In contrast, CHIR release was unaffected by the presence of RBV when co-loaded into the HG as RBV cannot bind via electrostatic interactions with CHIR or form extended hydrophobic associations. Taken together, these data suggest that varying drug combinations might be further exploited to ensure long term sustained release from PECE HGs, which have an intrinsic amphiphilicity. The combination of CHIR with PG545 or RBV in PECE HGs enables a dual-phase drug release system characterised by an initial burst release followed by sustained drug release lasting for at least 8 weeks. The burst release of RBV is attributed to high hydrophilicity, which results in enhanced partitioning into the aqueous release media. These properties are advantageous to target residual disease cells post-surgery via burst release and subsequently to impede tumour regrowth via sustained release prior to additional chemotherapy or radiotherapy. Additionally, the HG ability to simultaneously load and deliver two distinct payloads opens avenues for co-delivery of a cytotoxic agent in combination with a molecular targeted therapeutic, potentially enhancing clinical efficacy by consideration of patient-stratified drug delivery.

Low IC50 values conferred by CHIR and PG545 (alone and in combination) against a G3 MB in vitro panel, and by CHIR and RBV (alone and in combination) against an AT/RT panel, highlight the potency of these chemotherapeutics as both single and dual treatments (Fig. 2a-b), which is retained upon incorporation and release from PECE HG (Fig. 2c-d). Importantly, the blank PECE HG was non-toxic following incubation with both cell lines for 72 h. The enhanced efficacy of the dual drug loadings compared to their single agent counterparts can be attributed to the release kinetics, which emphasise the advantages of dual loaded HGs in the treatment of G3 MB and AT/RT in vitro.

Injection of PECE HG into the brains of healthy mice resulted in no significant decrease in animal weight over 15 days (Fig. 2e), with no observable apoptosis, proliferation or inflammation near the injection site (Fig. 2f). Collectively, these results indicate that the blank PECE HG at 20% (w/w) was well tolerated in the brain and did not result in any adverse effects. Against the D425 model, drug loaded HG was well tolerated with no reduction in animal body weight (Fig. 3b). Furthermore, the drug loaded HG when combined with XRT suggests a potential overall survival benefit as compared to the XRT alone treated group (p < 0.01) (Fig. 3a); however, low sample size precludes any inference of a statistically significant overall survival benefit. Nevertheless, it is of note that one LTS associated with a disease-free brain was evident in the drug loaded HG and XRT treatment group at the termination of the study. The current standard treatment for children diagnosed with G3 MB involves surgical resection and adjuvant chemotherapy and XRT [3], although systemic chemotherapy can result in severe side-effects which can have a lifelong impact on quality of life [38]. Local delivery of chemotherapeutics can potentially circumvent these issues, and our drug loaded PECE HG was not only well tolerated, but demonstrated enhanced efficacy when combined with XRT, indicating its potential for future clinical application against G3 MB tumours.

Against the BT12 model, drug loaded HGs were well tolerated (Figure S7), and drug loaded HG at 30% resulted in a significant increase in overall survival when compared to the control group (p < 0.0232) (Fig. 4b). A comparable overall survival was observed between the XRT and the 30 and 50% drug loaded HG treated groups. Additionally, there was one LTS present in both HG treated groups with the LTS in the 30% group deemed disease free, as determined by neuropathological microscopy review. Of note, whereas high Ki67 expression was evident for the one LTS in the 50% group, the observable tumour was clearly circumscribed (determined by post-mortem histology), suggesting impairment of tumour infiltration may be attributable to long-term survival in this animal. In a clinical setting to treat patients diagnosed with AT/RT, XRT is typically not recommended for young children < 3 years old due to adverse neurological effects and impact on quality of life in later years [39]. We therefore opted to determine the efficacy of our drug loaded HG system without adjuvant XRT to establish a clinically relevant treatment [40]. The comparable survival outcome between drug loaded HG-treated and XRT treated animals is encouraging, particularly for patients who are not eligible for XRT treatment.

It is important to note that due to the challenges to safe surgical resection in the mouse cerebellum, our study has assessed tolerability and efficacy against tumours xenografted into the mouse cortex. Future steps towards clinical translation will require validation in rat immunodeficient models with tumours xenografted into the cerebellum. Furthermore, for clinical translation of this LDDS, a thorough understanding of paediatric pharmacokinetics, neurodevelopment, and immune responses will be essential to minimize concerns regarding drug diffusion leading to off-target effects, dosing challenges due to variable metabolic rates, disruption of neurodevelopmental pathways and mechanical/structural tissue damage. Long-term studies and careful risk–benefit assessments are critical before widespread clinical use.

## Conclusions

This study has demonstrated a versatile drug delivery system with properties advantageous for post-resection therapeutic indications, including injectability, in situ gelation and compatibility of diverse drug types with no evidence of adverse effects. The dual release kinetics for two therapeutic agents (burst release in combination with sustained release), supports consideration of this LDDS as a platform technology for the treatment of G3 MB and AT/RT. Specifically, our work lays the foundation for flexible adaptation of a delivery system which can be loaded with a chosen cytotoxic drug exhibiting burst release, to be combined with a rationally selected molecular targeted therapeutic with a sustained release profile, predicated on genomic subtype-specific data, and in so doing, providing a route to ‘personalised drug delivery’.

## Supplementary Information

Below is the link to the electronic supplementary material.

ESM1 (DOCX 5.27 MB)

## Data Availability

The datasets generated during and/or analysed during the current study are available from the corresponding author on reasonable request.

## Declarations

### Animal studies

In vivo tolerability studies were conducted at Aix-Marseille Université and were approved by the Animal Care and Use Committee (CE71, Aix-Marseille Université) and performed following the French national regulation guidelines in accordance with EU Directive 2010/63/EU. Mice were housed in enriched cages placed in a temperature- and hygrometry-controlled room, had free access to water and food and were monitored daily. In vivo safety and efficacy studies were conducted at Johns Hopkins Medical School, Baltimore, US. All mice were treated in accordance with the Johns Hopkins University Care and Use Committee using an approved protocol.
