# Supplementary material for: Intraoperative drug delivery to hindbrain tumours via an injectable hydrogel is well tolerated and confers survival benefit against atypical teratoid/rhabdoid xenografts
Source: Drug Deliv Transl Res. 2026 Jan 4;16(7):2456–73. doi: 10.1007/s13346-025-02034-0 (PMC13294321; doi:10.1007/s13346-025-02034-0)
Supplement: Supplementary file 1 — (DOCX 5.27 MB) [file 13346_2025_2034_MOESM1_ESM.docx]

**Intraoperative drug delivery to hindbrain tumours via an injectable hydrogel is well tolerated and confers survival benefit against medulloblastoma and atypical teratoid/rhabdoid xenografts**

Cara Moloney^1,2^, Phoebe McCrorie^1^, Amr ElSherbeny^2,3^, Harry Porter^1^, Chiara Bastiancich^4^, Hasan Slika^5^, Aanya Shahani^5^, Emre Derin^5^, Esteban Velarde^6^, Jackson Miller^5^, John Theodore^5^, Khushi Varshney^5^, Hulya Bayraktutan^2^, Umut Can Oz^2,7^, Pam Collier^1^, Simon M. L. Paine^8^, Paul Handley^9^, Keith Dredge^9^, Grzegorz Wicher^10^, Richard G. Grundy^1^, Henry Brem^5^, Karin Forsberg-Nilsson^10,11,12^, Stuart J Smith^1^, Betty Tyler^5^, Cameron Alexander^2^, Ruman Rahman^1^

^1^ Children’s Brain Tumour Research Centre, School of Medicine, Biodiscovery Institute, University of Nottingham, UK.

^2^ Division of Molecular Therapeutics and Formulation, School of Pharmacy, University of Nottingham, UK.

^3^ Ex Vivo Cancer Pharmacology Centre of Excellence, School of Medicine, University of Nottingham, UK.

^4^ Aix-Marseille Université, Marseille, France.

^5^ Department of Neurosurgery, Johns Hopkins University, Baltimore, Maryland, USA.

^6^ Department of Radiation Oncology, Johns Hopkins University, Baltimore, Maryland, USA.

^7^ Department of Pharmaceutical Technology, Faculty of Pharmacy, Ankara University, Yenimahalle, Ankara, 06560 Türkiye.

^8^ Department of Neuropathology, Nottingham University Hospitals Trust, UK.

^9^ Zucero Therapeutics Ltd., Suite 1.11, Westlink Court, Darra, Queensland, Australia.

^10^ Department of Immunology, Genetics and Pathology and Science for Life Laboratory, Uppsala University, Uppsala, Sweden.

^11^ Science for Life Laboratory, Uppsala University, Uppsala, Sweden.

^12^ Biodiscovery Institute, School of Medicine, University of Nottingham, UK.

Corresponding authors:

Ruman Rahman - [mgzrr@exmail.nottingham.ac.uk](mailto:mgzrr@exmail.nottingham.ac.uk)

Cara Moloney - [mszcm3@exmail.nottingham.ac.uk](mailto:mszcm3@exmail.nottingham.ac.uk)

**Figure S1.** Rheological evaluation of PECE hydrogel prepared at 20% (w/v), loaded with **(a)** CHIR and PG545 or **(b)** CHIR and RBV at 1.5% (w/w) of each drug, showing effect of drug loading on the storage and loss modulus.

**Figure S2.** Scanning electron microscopy (SEM) images of PECE hydrogels, indicating the formation of porous structure. Representative SEM image of PECE hydrogel prepared at 20% (w/v) taken at varying magnification: **(a)** 0.85 kX, scale bar = 10 µm; **(b)** 2 kX, scale bar = 10 µm; **(c)** 9.5 kX, scale bar = 1 µm; and **(d)** 43 kX, scale bar = 100 nm.

**Figure S3.** Representative HPLC chromatogram for **(a)** a CHIR standard of 1.25 µg/mL, highlighting a retention time at 10.306 minutes; and **(b)** a RBV standard of 10 µg/mL, highlighting a retention time at 1.789 minutes.

**Figure S4.** Recorded cumulative release values and zoomed in release plots, respectively for CHIR **(a)** and **(b)**, PG545 **(c)** and RBV **(d)** and **(e)** from single or dual drug loaded PECE HGs. Release was recorded from 50 µL HG aliquots loaded at concentrations of 1.5% (w/w) of each drug and incubated in PBS (pH = 7.4) at 37°C (*n*=3, release reported as average ± S.D).

**Figure S5.** Recorded IC50 plots for single and dual treatments against the **(a)** D425, and **(b)** BT12 cell lines following 72-hour incubations. Recorded IC50 of **(c)** CHIR and PG545 against a panel of MB3 cell lines; and **(d)** CHIR and RBV against a panel of AT/RT cell lines.

**Figure S6.** Recorded average change (%) in tumour burden of mice implanted with D425 MB3 cells, which following surgical resection were treated with 10 Gy XRT (*n*=3), 10 µL of PECE loaded with CHIR/PG545 at 50% (w/w) with (*n*=6) or without (*n*=5) the addition of 10 Gy XRT (mean ± S.D).

**Figure S7.** **(a)** Recorded average change in mass of mice that were implanted with BT12 xenografts and following surgical resection were treated with: no treatment (control), 10 µL of blank PECE (20 % w/v), 10 Gy XRT, 10 µL of PECE loaded with (CHIR + RBV) at 30 or 50 % (w/w). **(b)** Corresponding recorded average tumour burden by IVIS imaging of fLuc tagged BT12 cells over the course of the experiment.

**Figure S8.** Histological evaluation of brain tissue of the longest survivor in each group following surgical resection of orthotopic AT/RT patient derived xenografts (BT12) and treatment. **(a)** Representative H&E staining of longest survivor in each treatment group highlighting the presence of recurrent tumour or resection cavity: **(i)** control day 127; **(ii)** control day 39; **(iii)** blank PECE day 44; **(iv)** 10 Gy XRT day 91; **(v)** PECE loaded with (CHIR + RBV) at 30 % (w/v) day 147 (LTS); and **(vi)** PECE loaded with (CHIR + RBV) at 50 % (w/v) day 147 (LTS). All images taken at 1.25X magnification, scale bar = 2.5 mm. **(b)** Quantification of the area occupied by recurrent BT12 tumours.
